# Subtype-specific shifts in age, axial length, and clinical profile of neovascular age-related macular degeneration: a five-year study in Japan

**DOI:** 10.1007/s10384-025-01302-3

**Published:** 2025-11-11

**Authors:** Masahiro Akada, Masayuki Hata, Midori Ideyama, Ai Kido, Manabu Miyata, Hiroshi Tamura, Sotaro Ooto, Akitaka Tsujikawa

**Affiliations:** 1 Department of Ophthalmology and Visual Sciences, Kyoto University Graduate School of Medicine, 54 Shogoin, Kawahara, Sakyo, Kyoto 606-8507 Japan; 2 Center for Innovative Research and Education in Data Science, Institute for Liberal Arts and Sciences, Kyoto University, Kyoto, Japan

**Keywords:** Neovascular age-related macular degeneration, Pachychoroid neovasculopathy, Axial length, Visual acuity outcomes, Longitudinal clinical trends

## Abstract

**Purpose:**

To evaluate 5-year temporal changes in baseline clinical characteristics—age, axial length, and best-corrected visual acuity—among treatment-naïve eyes with neovascular Age-Related Macular Degenaration (nAMD), comparing pachychoroid neovasculopathy (PNV) with drusen-driven (non-PNV) nAMD at a Japanese tertiary center.

**Study design:**

Retrospective observational study.

**Methods:**

Registry data from Kyoto University Hospital were analyzed for patients newly diagnosed with nAMD in 2013/2014 and in 2018/2019. Patients were classified as PNV or non-PNV based on findings derived from multimodal imaging—including optical coherence tomography, indocyanine-green angiography, and color fundus photography. Demographic data, axial length, best-corrected visual acuity (BCVA) at baseline and 1 year posttreatment, and the proportion of eyes achieving ≥0.20 logMAR improvement were compared over time.

**Results:**

A total of 118 patients were included. In the non-PNV group, mean age rose from 74.35 ± 8.42 years to 77.39 ± 7.90 years (p = 0.021), whereas the PNV group showed a smaller, non-significant change from 68.88 ± 7.25 to 70.41 ± 9.19 years (p = 0.48). Among non-PNV cases, both mean age (p=0.021) and axial length (p=0.017) increased significantly over time. In contrast, PNV cases showed no significant changes in age or axial length. BCVA outcomes and the proportion of patients achieving ≥0.20 logMAR improvement were similar across time points within each subtype. Multivariable logistic regression analysis revealed no significant associations between visual improvement and year, subtype, age, or axial length.

**Conclusions:**

This study revealed an aging trend and axial elongation among non-PNV cases over time, underscoring a subtype-specific divergence in clinical trajectory.

**Supplementary Information:**

The online version contains supplementary material available at 10.1007/s10384-025-01302-3.

## Introduction

Age-related macular degeneration (AMD) is a prototypical ocular aging-related disorder and a leading cause of vision loss among older adults in developed countries [1]. Among its subtypes, neovascular AMD (nAMD) is characterized by rapid progression and significant visual deterioration, often requiring repeated intravitreal injections of anti-vascular endothelial growth factor (anti-VEGF) agents [2]. These demands place a substantial burden on both patients and healthcare systems.

In Japan, which has one of the fastest-aging populations globally [3], the impact of nAMD on public health is considerable. Despite its recognition as a major public health issue, only limited epidemiological data are available on the trends in nAMD among older Japanese adults over time [4–8]. Furthermore, longitudinal evaluations of how the clinical characteristics of patients with nAMD have changed over time in actual practice remain limited [9–11], particularly with respect to disease subtypes.

To address this knowledge gap, we analyzed hospital-based registry data from Kyoto University Hospital to evaluate changes in the clinical profiles of patients diagnosed with nAMD between two pre-pandemic periods: 2013–2014 and 2018–2019. The availability of detailed ocular parameters, such as axial length, visual acuity, and subtype classification, enabled a focused investigation of temporal trends, including potential differences between pachychoroid neovasculopathy (PNV) and drusen-driven (non-PNV) AMD [12–16]. This study aims to provide real-world evidence of evolving nAMD patterns in a clinical setting within a super-aged society.

## Methods

### Study Design and Ethical Approval

This retrospective, single-center, observational study was approved by the Institutional Review Board and Ethics Committee of Kyoto University Hospital and Kyoto University Graduate School of Medicine (No. R0532). All procedures adhered to the principles of the Declaration of Helsinki and its subsequent amendments. Written informed consent was obtained from all participants in the hospital registry.

### Data Source and Study Population

Clinical data were obtained from Kyoto University Hospital, a tertiary referral center in Japan that routinely collects detailed ophthalmological parameters. Patients newly diagnosed with nAMD in 2013 or 2014 (2014 group) and in 2018 or 2019 (2019 group) were identified through electronic medical records. The inclusion criteria were age >50 years, axial length <26.5 mm, and the presence of treatment-naïve nAMD. Our cohort was assembled to include treatment-naïve eyes that began therapy with aflibercept injection. Eyes with any prior intraocular anti-VEGF therapy or photodynamic therapy (PDT) were excluded. Only one eye per patient was included in the study, and the other eye was not considered, even if it developed AMD. The exclusion criteria were previous treatment for macular neovascularization (MNV) and the presence of other retinal diseases, such as retinal vein or artery occlusion, diabetic retinopathy, myopic MNV, angioid streaks, and vitelliform macular dystrophy. Patients with chronic AMD, indicated by their disease history and/or massive fibrotic lesions, were also excluded. Those who dropped out of the study were excluded from the analysis. Each participant had received three monthly injections followed by four injections of aflibercept (2.0 mg) administered at two-month intervals during the first year of treatment.

### Variables and Definitions

For all included patients, we extracted information on age, sex, axial length, subfoveal choroidal thickness (SFCT), and best-corrected visual acuity (BCVA) at baseline and 1 year after anti-VEGF therapy initiation. SFCT was measured using enhanced depth imaging optical coherence tomography (EDI-OCT) in both horizontal and vertical scans, and the mean of the two values was used for analysis. The nAMD subtypes were classified as PNV or non-PNV (drusen-driven nAMD) based on multimodal imaging. The definition of PNV was based on previous studies [17–19] and included the following criteria: (1) presence of MNV in either eye; (2) clinical features of the pachychoroid phenotype, such as reduced fundus tessellation on fundus photographs, choroidal vascular hyperpermeability on indocyanine green angiography (ICGA), and dilated choroidal vessels on optical coherence tomography (OCT) and ICGA; and (3) No- or only non-extensive hard drusen in either eye. Polypoidal choroidal vasculopathy and type 2 MNV were classified according to their background phenotype: eyes that met the pachychoroid criteria were assigned to the PNV group, whereas eyes lacking these features were allocated to the non-PNV group. Diagnoses were made by two ophthalmologists, and in cases of discrepancy, a senior retinal specialist determined the final diagnosis. In the event of discordance, both graders reviewed the images together with a senior retinal specialist, and consensus was reached by joint inspection.

Data were analyzed separately for each subtype to assess potential differences in temporal trends. To evaluate the factors associated with clinically meaningful visual improvement, we conducted a multivariable logistic regression analysis. Visual improvement was defined as a decrease of ≥0.20 logMAR from baseline to 1 year. Independent variables included calendar year (2019 vs. 2014), AMD subtype (PNV vs. non-PNV), age, and axial length.

### Statistical Analysis

All analyses were performed using R (version 4.1.2; R Foundation for Statistical Computing) and Python version 3.11 (Python Software Foundation). Continuous variables were summarized as mean ± standard deviation and compared between the 2014 and 2019 groups using Student’s t-test or Welch’s t-test, depending on variance homogeneity. Changes in visual acuity within the same individual were evaluated using paired t-tests. Categorical variables were compared using the chi-square test or Fisher’s exact test, as appropriate. Statistical significance was set at a two-sided p-value <0.05.

## Results

### Changes in Patient Characteristics

After excluding six eyes that could not be followed after the initial visit due to systemic illness or personal circumstances (baseline characteristics summarized in Supplementary Table 1), a total of 118 newly diagnosed patients with nAMD at the Kyoto University Hospital were included in this study (Table 1). No eye underwent PDT during the follow-up. No significant changes were observed in the sex distribution of patients between the two time points. The mean age of patients at diagnosis increased from 73.24 ± 8.46 years in 2014 to 75.11 ± 8.92 years in 2019 (p = 0.117). Axial length also increased significantly from 23.36 ± 0.98 mm to 23.71 ± 1.10 mm (p = 0.018). When stratified by age (<75 vs ≥75 years), axial length increased over time in both groups, reaching statistical significance in patients aged <75 years (from 23.56 ± 0.96 mm to 24.01 ± 1.12 mm; p = 0.029), but not in those aged ≥75 years (23.14 ± 0.96 mm to 23.46 ± 1.03 mm; p = 0.089) (Supplementary Tables 2–3). BCVA significantly improved after treatment in each subtype in both groups.

### Subtype-Specific Trends

**Fig. 1:**
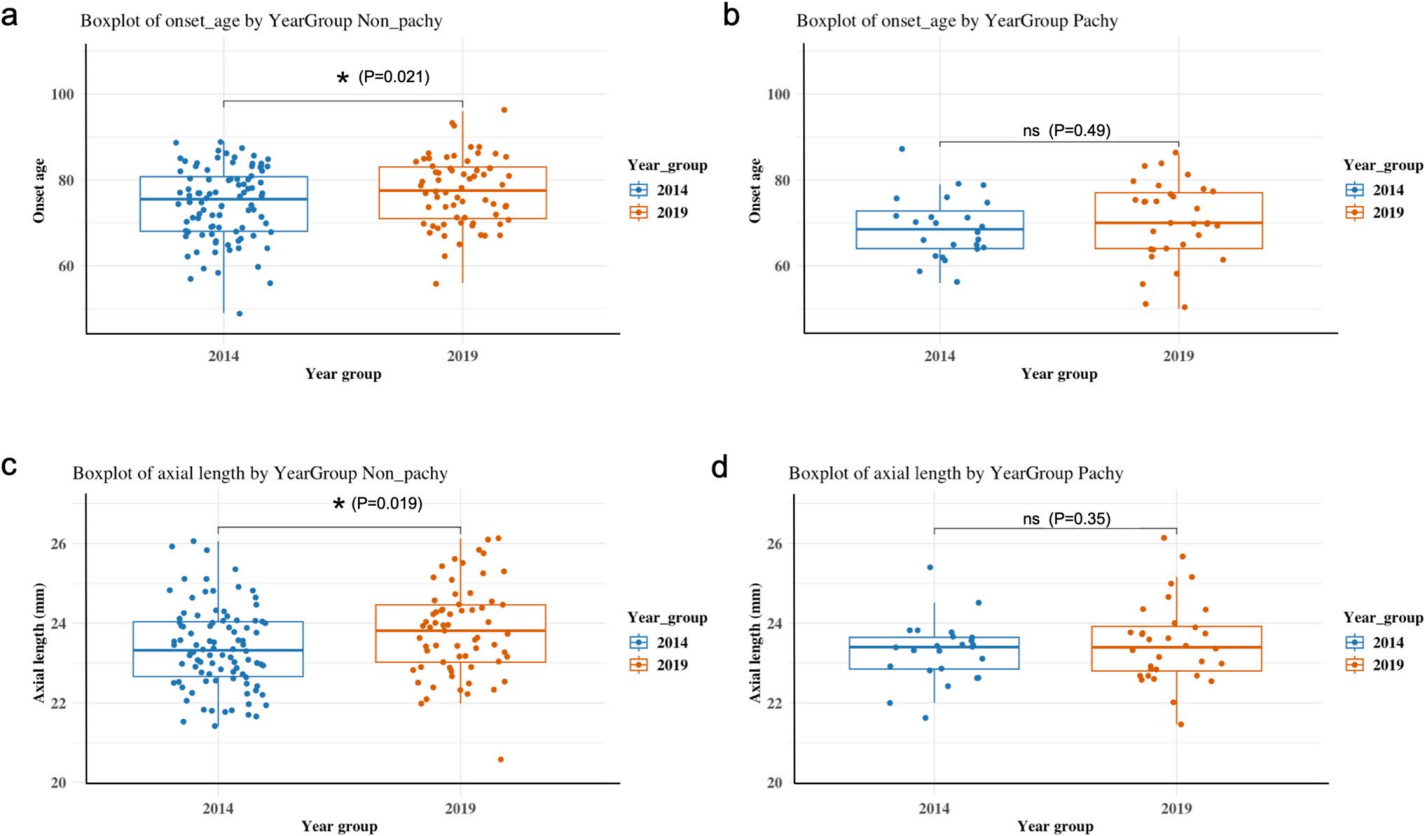
Boxplots comparing key ophthalmic parameters between 2014 and 2019, stratified by pachychoroid and non-pachychoroid nAMD. Boxplots comparing the age at onset and axial length between patients diagnosed in 2014 (blue) and 2019 (orange), stratified by pachychoroid status The total number of cases included was: overall (n = 118 in 2014, n = 98 in 2019), pachychoroid (n = 24 in 2014, n = 32 in 2019), and non-pachychoroid (n = 94 in 2014, n = 66 in 2019). a) In the non-pachychoroid nAMD group, the age at onset was significantly higher in 2019 than in 2014. b) In the pachychoroid nAMD group, no significant difference in age at onset was observed between the 2 years. c) In the non-pachychoroid group, the axial length was significantly longer in 2019 than in 2014. d) In the pachychoroid group, the axial length remained stable between 2014 and 2019 with no significant difference. The asterisk denotes statistical significance (p < 0.05); "ns" indicates a non-significant difference

**Fig. 2:**
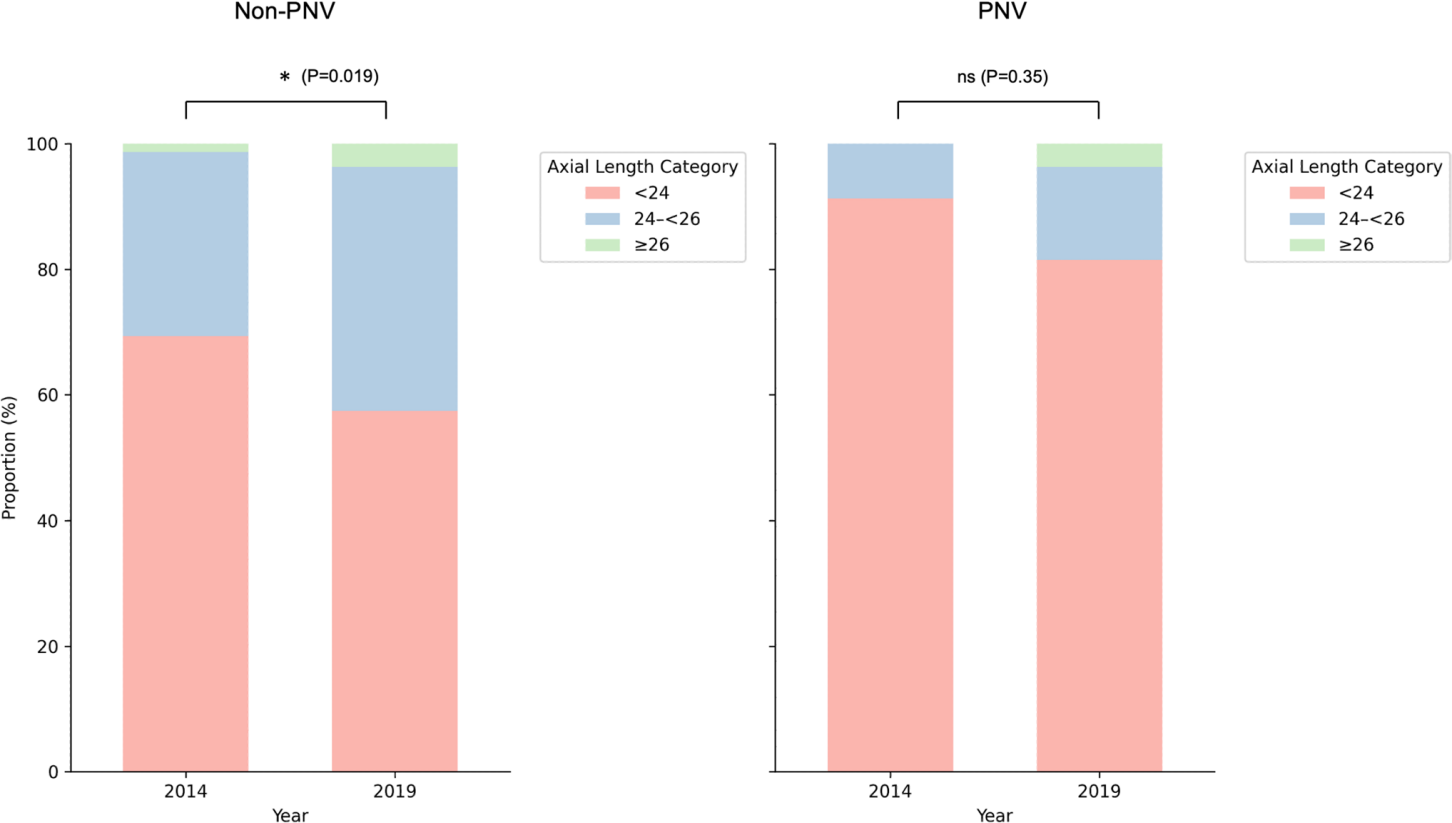
Axial length category distribution by subtype and year. The proportion of patients stratified by axial length categories (<24 mm, 24–<26 mm, ≥26 mm) in 2014 and 2019 is separately shown for non-PNV (n = 94 in 2014, n = 66 in 2019) and PNV (n = 24 in 2014, n = 32 in 2019) cases. In the non-PNV group, the axial length was significantly longer in 2019 than in 2014, whereas in the PNV group the axial length remained stable between 2014 and 2019 with no significant difference.

Subtype analyses revealed distinct trends between PNV and non-PNV (drusen-driven) nAMD (Table 1, Fig. 1–2). Combining data from both years, the axial length was slightly shorter in the PNV group (23.42 ± 0.93 mm) compared to the non-PNV group (23.55 ± 1.09 mm), though the difference was not statistically significant (p = 0.38). Among non-PNV cases, both mean age (74.35 ± 8.42 years in 2014 vs. 77.39 ± 7.90 years in 2019, p = 0.021) and axial length (23.38 ± 1.03 mm vs. 23.80 ± 1.13 mm, p = 0.019) showed statistically significant increases. When stratified by age (<75 vs ≥75 years), axial length increased over time in both age groups, with a statistically significant elongation observed in non-PNV patients aged <75 years (p = 0.019) (Supplementary Tables 2–3). In contrast, PNV cases demonstrated no significant changes in patient age (68.88 ± 7.25 years vs. 70.41 ± 9.19 years, p = 0.49), axial length, or BCVA during the study period.

### Visual Outcomes

**Fig. 3:**
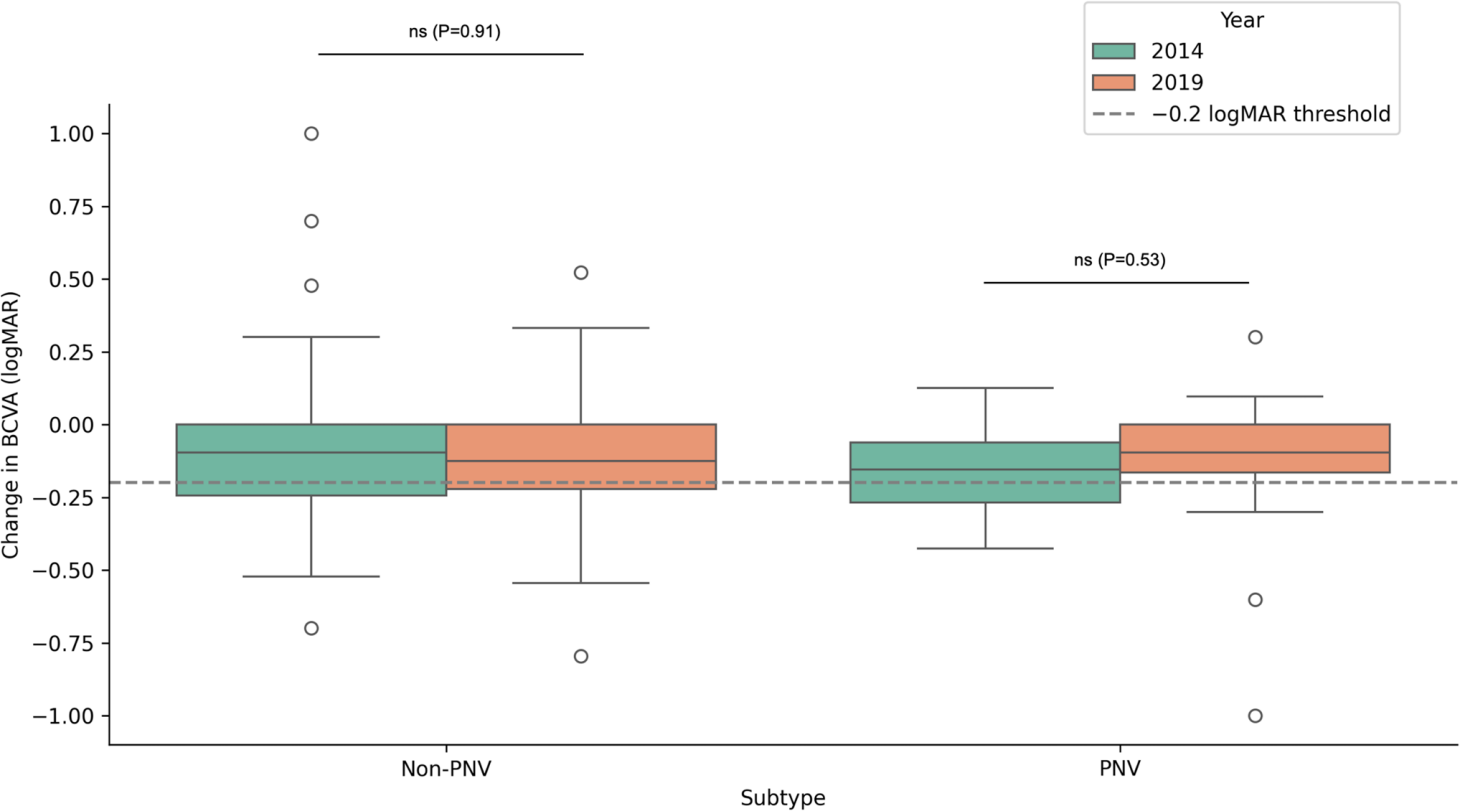
Change in BCVA (Posttreatment minus baseline) by subtype and year. Box plots showing the distribution of change in best-corrected visual acuity (BCVA, logMAR) from baseline to 1 year, stratified by subtype (non-PNV vs. PNV) and year (2014 vs. 2019). Negative values indicate visual improvement. The dashed line represents the threshold for meaningful improvement (Δ ≤ –0.2 logMAR). The median changes and interquartile ranges were comparable across years within each subtype, suggesting stable treatment outcomes over time. The numbers of cases were: non-PNV – 2014 (n = 94), 2019 (n = 66); PNV – 2014 (n = 24), 2019 (n = 32). PNV: pachychoroid neovasculopathy BCVA: best corrected visual acuity

Although posttreatment BCVA improved significantly across all subtypes and year groups, baseline BCVA, posttreatment BCVA, and degree of improvement did not differ significantly between the two periods in either the PNV or non-PNV groups. (Table 1, Fig. 3). Table 2 shows the results of the multivariable logistic regression analysis using visual improvement (defined as a ≥0.20 logMAR decrease at 1 year) as the dependent variable. Neither calendar year (OR = 0.77, 95% CI: 0.39–1.51, p = 0.443) nor AMD subtype (PNV vs. Non-PNV; OR = 0.60, 95% CI: 0.26–1.38, p = 0.232) was significantly associated with the likelihood of achieving visual improvement. Age (OR = 0.98 per year, 95% CI: 0.94–1.02, p = 0.364) and axial length (OR = 0.81 per mm, 95% CI: 0.58–1.12, p = 0.204) were also not significantly associated with the outcome.

## Discussion

In this study, we investigated changes in the clinical characteristics of patients with nAMD over a 5-year period in Japan. To minimize bias arising from differences in treatment regimens, we focused on two time points (2013/2014 and 2018/2019) during which anti-VEGF therapy at our institution remained relatively uniform (aflibercept). This approach allowed for a more consistent comparison by avoiding heterogeneity introduced by newer agents and evolving treatment paradigms adopted in subsequent years. Between the year groups, we observed a significant increase in both patient age and axial length, particularly among patients with drusen-driven (non-PNV) nAMD. Our previous nationwide claims database study reports an increase in the age of newly diagnosed patients with nAMD over time [20]. With detailed imaging-based phenotyping including subtype assignment, our present hospital-based findings align with this observation and further reveal subtype-specific trends, complementing our prior nationwide claims study. These findings suggest a gradual epidemiological shift in the clinical profile of patients with nAMD, potentially reflecting broader demographic and refractive trends in the Japanese population.

In PNV, the axial length tends to be shorter than in non-PNV, and previous studies report an inverse correlation between axial length and choroidal thickness [17, 21, 22]. This may be explained by increased venous outflow resistance in eyes with a short axial length, which could contribute to PNV pathogenesis [23]. In our cohort, eyes with PNV showed a trend toward longer axial length and thinner choroid in 2018/2019 compared to 2013/2014, although not statistically significant. At the same time, we observed a significant increase in axial length over time in the non-PNV group. While a shorter axial length has previously been associated with early AMD, particularly in meta-analyses of population-based studies, its relevance in late-stage or active nAMD remains unclear [24]. Although a direct relationship between AMD and myopia remains uncertain, the observed increase in axial length, especially among patients in the non-PNV group, aligns with the population-level trend of increasing myopia in East Asia [25–29]. Our age-stratified analysis indicates elongation in both younger and older patients (with statistical significance in those <75 years), supporting the view that this trend is not explained solely by an overall increase in mean age. In clinical practice, a longer axial length may be associated with choroidal thinning and lesion morphology characteristic of myopic eyes. These anatomical characteristics may influence treatment responsiveness and prognosis. Accurate assessment of macular atrophy and its progression may become more critical, underscoring the importance of early therapeutic intervention.

Despite these demographic and anatomical changes, visual outcomes remained stable over time. No significant differences were found in baseline or posttreatment BCVA, and the proportion of eyes achieving a ≥0.20 logMAR improvement was comparable between years. Multivariable logistic regression analysis confirmed that calendar year, subtype, age, and axial length did not significantly predict the likelihood of visual improvement. These findings suggest that demographic shifts do not have a substantial impact on treatment outcomes, although subtle changes in response patterns warrant further investigation.

This study has several strengths. The study leverages detailed multimodal imaging-based subtype classification and ophthalmic biometric data, such as axial length and BCVA. The subtype-specific analysis allowed us to distinguish between clinical shifts that would otherwise have been masked in the aggregate data. However, this study also has several limitations. First, as a single-center study with a relatively small sample size, findings may not be generalizable to the entire Japanese population. In particular, the small number of PNV cases may have limited our ability to detect significant differences in axial length or age. PNV is reported to be more common in men in Japan compared to Western countries [30–32]; still, the proportion of male cases was particularly high in this study. Furthermore, the observed rise in the proportion of PNV cases may be influenced by increased detection through multimodal imaging and evolving referral patterns, and, therefore, cannot be interpreted as a nationwide population-level trend. Second, although diagnosis and classification were confirmed by retinal specialists using multimodal imaging, potential interobserver variability cannot be entirely excluded. Because consensus grading was applied to the entire dataset, the frequency of initial discordance was not recorded, which represents a methodological limitation. Third, selection bias may exist as patients treated at a tertiary care center may not reflect the full spectrum of nAMD severity in the community. It also remains unclear whether the observed increase in patient age reflects disease-specific changes or demographic aging in Japan. In addition, the six dropouts may have introduced additional bias.

In conclusion, this hospital-based longitudinal analysis suggests that the clinical profile of nAMD, particularly in drusen-driven cases, is gradually shifting toward older age and longer axial length in Japan. These findings provide novel insights into subtype-specific epidemiological trends in nAMD and highlight the importance of continuous monitoring of demographic and biometric changes in clinical practice.

## Supplementary Information

Below is the link to the electronic supplementary material.

Supplementary file1 (PDF 189 KB)

## Data Availability

The datasets analyzed in the current study are not publicly available because of institutional data-sharing restrictions.

## Code availability

The code used in the analysis is available from the corresponding author upon request.
