# Supplementary material for: Subtype-specific shifts in age, axial length, and clinical profile of neovascular age-related macular degeneration: a five-year study in Japan
Source: Jpn J Ophthalmol. 2025 Nov 11;70(3):572–8. doi: 10.1007/s10384-025-01302-3 (PMC13226323; doi:10.1007/s10384-025-01302-3)
Supplement: Supplementary file 1 — Supplementary file1 (PDF 189 KB) [file 10384_2025_1302_MOESM1_ESM.pdf]

Supplemental Table 1. Baseline Characteristics of Patients Who Dropped Out After Initial Visit

Year	Sex	Subtype	Axial length (mm)	Age	BCVA (logMAR)	SFCT (μm)
2014	Female	Non-PNV	22.7	81	0	166
2018	Male	Non-PNV	23.5	84	0.15	178
2018	Male	Non-PNV	24.1	84	0.30	276
2018	Male	Non-PNV	24.6	69	0.69	154
2018	Male	Non-PNV	26.2	77	0	137
2019	Male	Non-PNV	24.5	67	0.82	103

Abbreviations: BCVA, best-corrected visual acuity; SFCT, sub-foveal choroidal thickness

Supplemental Table 2. Characteristics of nAMD patients Aged <75 in 2014 and 2019

Variable	Overall 2014	Overall 2019	*p*-value	Non-PNV 2014	Non-PNV 2019	*p*-value	PNV 2014	PNV2019	*p*-value
**Total Cases**	63	44		45	26		18	18	
**Age, years, mean (SD)**	66.67 (5.23)	67.20 (5.88)	0.620	67.09 (5.48)	69.42 (4.11)	0.064	65.61 (4.50)	64.00 (6.66)	0.401
**Male:Female (n)**	48 (76.2%) : 15 (23.8%)	28 (63.6%) : 16 (36.4%)	0.233	33 (73.3%) : 12 (26.7%)	15 (57.7%) : 11 (42.3%)	0.274	15 (83.3%) : 3 (16.7%)	13 (72.2%) : 5 (27.8%)	0.688
**Axial Length (SD)**	23.56 (0.96)	24.01 (1.12)	0.029	23.64 (1.00)	24.25 (1.05)	0.019	23.35 (0.87)	23.67 (1.16)	0.357
**SFCT (SD)**	267.92 (86.91)	262.32 (90.92)	0.749	241.39 (78.80)	204.29 (52.95)	0.036	338.15 (67.06)	346.14 (64.80)	0.722
**BCVA before treatment (SD)**	0.30 (0.34)	0.30 (0.41)	0.989	0.31 (0.37)	0.33 (0.39)	0.856	0.26 (0.23)	0.25 (0.45)	0.967
**BCVA after treatment (SD)**	0.16 (0.36)	0.18 (0.35)	0.883	0.20 (0.41)	0.24 (0.31)	0.651	0.10 (0.23)	0.08 (0.39)	0.832
**Change in BCVA (SD)**	-0.14 (0.24)	-0.14 (0.26)	0.909	-0.13 (0.28)	-0.10 (0.26)	0.657	-0.15 (0.15)	-0.21 (0.25)	0.402
**p-value for pre- vs. post-treatment BCVA***	<0.001	0.0013		0.0110	0.0793		<0.001	0.00475	

* P-values represent within-group paired comparisons of BCVA before vs. after treatment (paired t test).

Abbreviations: SFCT, sub-foveal choroidal thickness; PNV, pachychoroid neovasculopathy; non-PNV, drusen-driven neovascular AMD.

Supplemental Table 3. Characteristics of nAMD patients Aged ≥75 in 2014 and 2019

Variable	Overall 2014	Overall 2019	*p*-value	Non-PNV 2014	Non-PNV 2019	*p*-value	PNV 2014	PNV2019	*p*-value
**Total Cases**	55	54		49	40		6	14	
**Age, years, mean (SD)**	80.76 (3.94)	81.56 (4.84)	0.350	81.02 (3.85)	82.58 (4.81)	0.094	78.67 (4.41)	78.64 (3.67)	0.990
**Male:Female (n)**	35 (63.6%) : 20 (36.4%)	31 (57.4%) : 23 (42.6%)	0.639	29 (59.2%) : 20 (40.8%)	23 (57.5%) : 17 (42.5%)	>0.99	6 (100.0%) : 0 (0%)	8 (57.1%) : 6 (42.9%)	0.166
**Axial Length (SD)**	23.14 (0.96)	23.46 (1.03)	0.089	23.14 (1.01)	23.52 (1.09)	0.098	23.09 (0.38)	23.31 (0.83)	0.546
**SFCT (SD)**	211.81 (91.66)	224.54 (97.49)	0.488	196.02 (82.67)	193.16 (89.55)	0.878	340.75 (52.32)	309.71 (61.42)	0.296
**BCVA before treatment (SD)**	0.41 (0.36)	0.40 (0.34)	0.835	0.43 (0.36)	0.41 (0.32)	0.794	0.28 (0.31)	0.37 (0.41)	0.642
**BCVA after treatment (SD)**	0.29 (0.36)	0.29 (0.35)	0.986	0.32 (0.37)	0.29 (0.32)	0.678	0.09 (0.25)	0.30 (0.44)	0.299
**Change in BCVA (SD)**	-0.09 (0.21)	-0.08 (0.21)	0.846	-0.08 (0.21)	-0.11 (0.22)	0.493	-0.19 (0.15)	0.00 (0.14)	0.022
**p-value for pre- vs. post-treatment BCVA***	0.00473	0.0158		0.0276	0.00909		0.0301	0.937	

* P-values represent within-group paired comparisons of BCVA before vs. after treatment (paired t test).

Abbreviations: SFCT, sub-foveal choroidal thickness; PNV, pachychoroid neovasculopathy; non-PNV, drusen-driven neovascular AMD.
